# Commentary: PCP4 inhibits the progression of prostate cancer through Ca^2+^/CAMKK2/AMPK/AR pathway

**DOI:** 10.3389/fimmu.2026.1875302

**Published:** 2026-07-03

**Authors:** Wujie Chen, Fajiang Qian, Fengtong Wang, Fuquan Yang, Yuqing Shen, Mingxia Ding, Yuan Liu

**Affiliations:** 1 Kunming Medical University, Kunming, China; 2 Department of Urology, The First People’s Hospital of Yunnan Province, Kunming, China; 3 Department of Urology, Lincang People’s Hospital Linxiang District, Lingcang, Yunnan, China; 4 Department of Oncology, Lincang People’s Hospital Linxiang District, Lingcang, Yunnan, China; 5 Medical Device Registration Production Inspection Section, China Food and Drug Administration, Medical Products Administration of Yunnan, Kunming, Yunnan, China; 6 Department of Orthopedics, Chongqing Qianjiang Central Hospital, Chongqing, China

**Keywords:** CAMKK2, castration-resistant prostate cancer, commentary, PCP4, prostate cancer

## 1 Introduction

We read with great interest the recent article by Jia and colleagues titled “PCP4 inhibits prostate cancer progression via the Ca^2+^/CAMKK2/AMPK/AR signaling pathway” (1). In this excellent study, the authors systematically investigated the role of Purkinje cell protein 4(PCP4) (2)in prostate cancer, particularly in castration-resistant prostate cancer (CRPC). Through public database mining, *in vitro* functional assays, *in vivo* xenograft models, and molecular mechanistic studies, they demonstrated that downregulation of PCP4, frequently caused by genetic deletion, promotes prostate cancer progression by activating the Ca^2+^/CAMKK2/AMPK/AR signaling axis. This work provides novel insights into the molecular mechanisms underlying CRPC and identifies PCP4 as a potential therapeutic target.

The treatment of CRPC remains a major challenge in urologic oncology. The study by Jia and colleagues not only offers a new molecular pathway for understanding CRPC progression but, more importantly, highlights PCP4 as a potential therapeutic target and prognostic marker. Through multi-layered experimental validation, the authors systematically elucidate how PCP4 loss stabilizes the CAMKK2 protein via activation of the Ca^2+^ signaling pathway, thereby enhancing the transcriptional activity of both AMPK and AR, forming a complete signaling cascade.

This finding carries significant clinical translational implications: the use of the CAMKK2 inhibitor STO-609 reversed the proliferative effect induced by PCP4 knockdown, suggesting that therapeutic strategies targeting the PCP4-CAMKK2 axis are feasible in PCP4-deficient CRPC. Furthermore, the strong negative correlation of PCP4 expression with T stage, Gleason score, and biochemical recurrence provides compelling evidence for its role as an independent prognostic factor.

We hold great admiration for the academic contributions of the research team. At the same time, we would like to offer a few constructive suggestions regarding several potentially extensible directions in this study, hoping to provide reference for future investigations.

## 2 Major academic contributions of this study

### 2.1 Comprehensive research framework and impressive multi-dimensional validation system

A distinctive strength of this study lies in its establishment of a complete chain of evidence spanning from molecular to animal to clinical data. The authors first identified differentially expressed genes through cross-analysis of multiple databases, then performed functional validation in CRPC cell models, extended the investigation to *in vivo* animal experiments, and finally focused on elucidating the molecular mechanisms underlying the Ca^2+^/CAMKK2/AMPK/AR signaling pathway. This research paradigm—”clinical data analysis →cellular functional validation → *in vivo* validation → mechanistic dissection”—not only substantially enhances the rigor and reliability of the study’s conclusions but also provides a methodological template for similar investigations. Notably, the authors validated key nodes of the signaling pathway through multiple complementary approaches, including protein degradation assays (CHX chase), Ca^2+^ chelation and release manipulation, and rescue experiments using a CAMKK2 inhibitor, reflecting a robust scientific reasoning process.

### 2.2 Clear clinical translational potential, offering new targets for precision therapy

The study systematically analyzed the association between PCP4 expression and clinicopathological parameters of prostate cancer patients by leveraging TCGA, SU2C, MSKCC, and multiple GEO datasets. Low PCP4 expression was significantly correlated with higher T stage, higher Gleason score, and markedly shorter DFS and BCRFS. Furthermore, the co-occurrence of PCP4 deletion and TMPRSS2-ERG fusion in CRPC carries important clinical implications—this provides a theoretical basis for patient stratification based on PCP4-ERG co-deletion status, representing a compelling illustration of precision medicine principles in the field of prostate cancer. As recently articulated in the “actionable transcriptome” framework proposed by the MD Anderson Cancer Center, integrating findings from the RNA expression level into clinical pathways to guide targeted and immunotherapeutic decisions has become an important direction in precision oncology (3).

### 2.3 Discovery of feedback regulation within the signaling pathway, advancing understanding of the AR network

This study is the first to definitively identify PCP4 as a repressive target gene of AR while also revealing a bidirectional feedback regulation between PCP4 and CAMKK2: PCP4 downregulation promotes CAMKK2 expression, whereas CAMKK2 knockdown in turn upregulates PCP4. The discovery of this feedback loop carries significant biological implications—it explains how persistent low expression of PCP4 and sustained high expression of CAMKK2 in CRPC can establish a self-maintaining positive feedback that drives irreversible tumor progression toward castration resistance. This concept finds supporting evidence in the CAMKK2-AR field, where a feedback loop maintaining receptor activity between CAMKK2 and AR has been described (4). The present study further complements the gatekeeper role of PCP4 in this context, enriching our understanding of aberrant AR activation in CRPC progression.

## 3 Suggestions for study optimization

Notwithstanding the notable strengths outlined above, we believe that several aspects of this study could be further refined to enhance mechanistic depth and conclusion robustness.

### 3.1 The molecular mechanism by which PCP4 regulates CAMKK2 protein stability remains to be elucidated

Using cycloheximide (CHX) chase assays, the authors demonstrated that PCP4 influences the half-life of CAMKK2—PCP4 downregulation slows, whereas PCP4 overexpression accelerates, CAMKK2 degradation. This finding opens new avenues for understanding PCP4 function. However, several critical questions remain unanswered: through which degradation pathway (proteasomal vs. autophagy-lysosomal) does PCP4 exert its effect? Are specific E3 ubiquitin ligases involved? And how does PCP4, a small peptide of approximately 7.6 kDa lacking intrinsic enzymatic activity, modulate CAMKK2 degradation? Notably, the presence of inhibitory motifs within PCP4 family proteins that regulate protein function suggests that PCP4 may mediate its regulatory effects through protein–protein interactions (5).

We propose that future studies could address these questions through the following strategies: A. treating cells with MG132 (proteasomal inhibitor), chloroquine (autophagy inhibitor), and 3-MA (autophagy initiation inhibitor) to determine the degradation pathway by comparing changes in PCP4-mediated CAMKK2 degradation across treatment groups; B. generating lysine-to-arginine mutant systems targeting ubiquitination sites on CAMKK2, coupled with immunoprecipitation-mass spectrometry (IP-MS) to screen for potential E3 ligases (e.g., CHIP, MDM2) or deubiquitinating enzymes involved; and C. employing molecular dynamics simulations or structural biology approaches to resolve the binding mode of PCP4 with the CAMKK2/CaM complex.

### 3.2 Functional validation is required for the co-deletion of PCP4 and TMPRSS2-ERG fusion

Database analyses revealed a co-occurrence tendency between PCP4 deletion and TMPRSS2-ERG fusion in prostate cancer. However, as both genetic events are located within the 21q22.2 chromosomal region, their observed association may simply reflect regional co-deletion rather than functional synergistic drive. Therefore, correlation analyses based solely on database mining are insufficient to establish a functional relationship between these two events. We recommend that future studies systematically evaluate whether PCP4 deletion and ERG fusion exert functional synergy in tumor progression through multiple approaches, including *in vitro* co-manipulation (e.g., combined gene knockdown and overexpression), *in vivo* dual genetic modification animal models, and stratified analyses of clinical cohorts. Such investigations would clarify whether these two events function as independent drivers or as cooperative factors in promoting CRPC progression.

### 3.3 Dynamic discrimination of Ca^2+^ signal origin requires further elucidation

The study employed BAPTA-AM to chelate intracellular Ca^2+^ and thapsigargin to inhibit the endoplasmic reticulum Ca^2+^ ATPase, providing preliminary validation of the intermediary role of Ca^2+^ signaling in PCP4-mediated regulation of CAMKK2. However, because BAPTA-AM indiscriminately chelates Ca^2+^ from all intracellular sources, this approach cannot distinguish whether Ca^2+^ elevation originates from endoplasmic reticulum Ca^2+^ release, extracellular Ca^2+^ influx, or both. Similarly, thapsigargin only depletes endoplasmic reticulum Ca^2+^ stores and fails to exclude potential additive effects from extracellular Ca^2+^ influx pathways mediated by calcium channels such as TRPV or Orai. This distinction is critical because Ca^2+^ from different sources often triggers distinct downstream effectors, thereby influencing tumor cell proliferation, migration, apoptosis, and other biological behaviors. For instance, dihydrotestosterone (DHT) has been reported to promote Ca^2+^ influx in prostate cancer cells via the TRPV6 channel (6). Future studies could systematically dissect the origin and dynamic characteristics of Ca^2+^ signals underlying PCP4-mediated CAMKK2 regulation by differentiating the relative contributions of extracellular Ca^2+^ influx versus intracellular Ca^2+^ release, screening for the types of calcium channels or transporters involved, and employing high spatiotemporal resolution Ca^2+^ dynamic monitoring techniques.

## 4 Methodological synthesis and broader implications—paradigm reflections from this study to similar tumor marker investigations

### 4.1 Paradigmatic significance of the PCP4 series: the evolution from a “housekeeping protein” to a “tumor-associated molecule”

PCP4 was originally identified in 1986 as a 7.6 kDa small polypeptide in rat cerebellar Purkinje cells, and for a prolonged period, research focused primarily on its calmodulin-modulating functions within the central nervous system (7). In recent years, however, accumulating evidence has revealed that PCP4 exhibits marked and functionally diverse expression profiles across multiple tumor types, demonstrating a high degree of functional plasticity—i.e., a context-dependent mode of action whereby its functional outcome depends on the specific microenvironment and molecular background of the tumor cell in question. Honjo and colleagues showed that in breast cancer, PCP4 upregulates aromatase expression via the CYP19A1 promoter I.1, thereby supplying estrogen within the tumor microenvironment (8). By contrast, Yoshimura et al. found that in mucoepidermoid carcinoma, PCP4 expression correlates with better prognosis, suggesting that PCP4 and HER2 could serve as combined prognostic markers for this disease (9).

Building upon this foundation, the study by Jia and colleagues further expands the functional repertoire of PCP4 in prostate cancer. In contrast to reports demonstrating an oncogenic role for PCP4 in breast cancer, the present study unequivocally reveals that PCP4 exerts a tumor-suppressive function in prostate cancer. This dichotomous—indeed, opposing—function of the same molecule across different cancer types suggests that the biological activity of PCP4 is profoundly influenced by its cellular context and the surrounding signaling network environment. Consequently, functional characterization of PCP4 must be performed in a cancer type-specific manner, taking into account the distinct mutational landscapes and microenvironmental features of each malignancy, rather than being generalized across tumors. As a marker that has “spilled over” from the brain to multiple cancer types, the research trajectory of PCP4 itself offers an important lesson: the screening of candidate tumor markers should broaden its horizons appropriately and not remain confined to traditionally recognized cancer-related genes. Even “housekeeping molecules” originating from the central nervous system may play significant roles in specific tumor types.

### 4.2 The database-driven “reverse biology” research paradigm: from big data to functional validation

Methodologically, this study elegantly exemplifies the emerging “reverse biology” paradigm in tumor molecular biology. The traditional research path typically follows a forward direction—”cellular functional observation → molecular mechanism identification → clinical correlation validation.” In contrast, the investigative trajectory adopted in this study—initiating with large-scale public database mining followed by backward validation through cellular and animal experiments—represents a complementary approach. Specifically, the study began with transcriptomic differences between CRPC and primary prostate cancer in the GEO database, employed cross-database overlapping analysis to pin down PCP4 as the key molecule, and finally confirmed its biological functions through *in vitro* functional assays and *in vivo* tumor formation models.

This “data-first, experiment-validation” strategy has increasingly become an efficient paradigm for tumor marker research. The EGNF framework published in 2025 by the MD Anderson Cancer Center further illustrates the evolution of this field. This method employs graph neural networks to construct dynamic, patient-specific molecular interaction networks between gene expression data and clinical attributes, enabling complete classification of tumor versus normal samples and precise differentiation of disease progression and treatment outcomes (10). Similarly, the GASPS framework from Baylor College of Medicine provides a systematic approach for patient prognostic stratification based on transcriptomic dysregulation signatures driven by genomic structural variants (11). The most striking insight from this framework is that while genetic mutations themselves have limited prognostic value, the transcriptomic signatures driven by these mutations possess substantially stronger prognostic predictive power. Therefore, for a molecule such as PCP4, which exhibits a relatively high (though not 100%) frequency of genetic deletion, integrating its deletion status with transcriptomic expression profiles to construct a PCP4 signaling intensity scoring system might yield clinically more valuable risk stratification.

### 4.3 The Ca^2+^/CaM signaling axis and its crosstalk with tumor metabolism/AR signaling: a modular research paradigm for a class of targets

At the mechanistic level, this study illustrates a highly representative tumor target module—the crosstalk between the Ca^2+^/CaM signaling axis and tumor metabolism/AR signaling. Jia and colleagues took PCP4, a dynamic factor regulating Ca^2+^/CaM binding, as the entry point and linked it to CAMKK2—a core kinase activated by Ca^2+^/CaM that directly influences cellular energy metabolism and AR transcriptional activity via p-AMPK(Thr172)—thereby constructing a complete signaling logic chain of “upstream Ca^2+^ regulator → downstream effector kinase → ultimate transcription factor.”

This research paradigm demonstrates strong transferability. As a central hub of the Ca^2+^/CaM signaling pathway, CAMKK2 has been shown in prostate cancer to be induced by AR while also maintaining AR stability in return, forming a positive feedback loop (4, 12). Within this loop, PCP4 acts as a “gatekeeper”—indirectly influencing CAMKK2 protein stability through its regulation of CaM binding. This mechanistic example illustrates the adaptability of the “core hub node plus regulatory node” research model. Investigators can focus on the core CAMKK2-AMPK-AR axis and, across different tumor types or therapeutic contexts (such as endocrine therapy resistance or metabolic reprogramming), search for novel upstream regulatory factors (e.g., specific miRNAs, E3 ligases, or CaM-binding proteins) and rapidly validate their functions by adopting the modular signaling detection system established in this study.

### 4.4 From “single-pathway investigation” to “networked integration”: implications for similar tumor marker studies

Although the present study achieves a clear and complete delineation of the Ca^2+^/CAMKK2/AMPK/AR signaling axis, from the perspective of general methodologies in tumor marker research, future investigations may consider extending the paradigm from a “node–pathway” approach toward “multi-dimensional network integration” in the following directions:

(1) Multi-omics integrative analysis. The current study primarily relied on transcriptomic data for molecular screening. Subsequent investigations could integrate genomic (CNV/mutation), proteomic, epigenetic, and metabolomic data to more comprehensively delineate the molecular landscape of tumor cells following PCP4 loss, thereby identifying additional potential key regulatory nodes and intervention targets.

(2) Mechanistic dissection at single-cell resolution. Downregulation of PCP4 expression does not occur synchronously across all tumor cells *in vivo* but rather exhibits a subclonal distribution pattern against the backdrop of tumor heterogeneity. Application of high-throughput single-cell RNA sequencing could be employed to precisely delineate transcriptomic differences between PCP4-deleted and non-deleted clones, to determine whether key signaling pathway alterations induced by PCP4 loss are confined to specific cellular subsets, and to elucidate how such heterogeneity influences the evolutionary trajectory of CRPC.

## 5 Conclusion

In summary, the study by Jia and colleagues is the first to systematically reveal the tumor-suppressive role of PCP4 in prostate cancer, particularly during CRPC progression, and to elucidate the molecular mechanism by which PCP4 loss drives malignant tumor progression through activation of the Ca^2+^/CAMKK2/AMPK/AR signaling axis. The scientific rigor of this study is particularly evident in four aspects: the multi-database-based molecular screening ensures breadth and representativeness; the combination of *in vitro* functional assays and *in vivo* animal models guarantees robust phenotypic validation; the multi-pronged approach incorporating protein degradation assays and Ca^2+^ signaling manipulation ensures mechanistic reliability; and the positive results from STO-609 inhibitor rescue experiments provide preliminary proof-of-concept for clinical translational feasibility. We firmly believe that this study not only establishes a solid theoretical foundation for the role of PCP4 family members in prostate cancer but also provides important preclinical evidence supporting the advancement of CAMKK2 inhibitors toward clinical therapeutic strategies for PCP4-deficient CRPC.

We sincerely hope that the suggestions proposed herein—elucidating the molecular pathway by which PCP4 regulates CAMKK2 degradation, validating the functional synergy between PCP4 deletion and ERG fusion, refining the discrimination of Ca^2+^ signal origins, and extending multi-omics integration and microenvironmental investigations—may serve as a source of inspiration for the authors and fellow researchers in the field. We once again thank the team led by Jia for their important academic contribution to this research area.
